# Clinical Lecture on Congenital Cataract, Delivered at the Birmingham Eye Infirmary, October 30, 1832

**Published:** 1833-06

**Authors:** 


THE LONDON

### Medical and Physical Journal.

412, VOL. LXIX.] JUNE 1833. [84, *New Series.*

"For many fortunate discoveries in medicine, and for the detection of numerous errors, the world is indebted to the rapid circulation of Monthly Journals; and there never existed any work to which the Faculty in Europe and America, were under deeper obligations than to the 'Medical and Physical Journal of London,' now forming a long but an invaluable series."—RUSH.

### ORIGINAL PAPERS.

### CONGENITAL CATARACT.

*Clinical Lecture on Congenital Cataract, delivered at the Birmingham Eye Infirmary, October* 30, 1832, by Mr. MIDDLEMORE.

GENTLEMEN: You are aware that infants are occasionally born with cataract in one or both eyes; they have what is termed congenital cataract; and I may mention it to you, that this disease sometimes occurs in several children of the same family: we have, for instance, seen at this institution four children, brothers or sisters, (I forget which,) all of whom were born with cataract in each eye, and you will find some information relating to this singular occurrence in the work of the late Mr. Saunders, p. 158, and in the excellent Lectures of Dupuytren. You will remember therefore that congenital cataract is, in some instances, a peculiarity connected with the development of a particular part of the eye, which may occasionally be noticed in various members of the same family. I cannot, however, find that the brothers or sisters of the young man whose case I shall presently relate, have suffered from any similar disease.

CASE. Thomas Gardner, twenty-two years old, has congenital cataract in each eye. His mother informed me that she took him to Dr. Power, of Lichfield, when he was very young, and that the doctor recommended that no operation should be performed until he was older, and, not wishing to *tamper* with the eyes, she had allowed the disease to remain.

Now, before I performed an operation upon him, he could just see to walk about; could sometimes, by placing a book almost in contact with his eyes, and looking at it laterally, make out letters; could distinguish suddenly-contrasted degrees of light, the more vivid colours, and so on; but he possessed no degree of vision which enabled him to read at all freely, or follow any ordinary employment.

*Appearance of the cataract.* A densely opaque membrane, rather smaller than the pupil, (which was naturally under the influence of an ordinary degree of light,) was situated immediately behind the posterior chamber; it was dotted, or marbled, and was evidently the capsule of the lens.

*Other circumstances connected with the state of the eye.* The eyeball is very small; its muscles have acquired an irritable unsteady action, and the globe is continually rolling about in the orbit. The cornea is exceedingly diminutive, and rather more convex than usual; the iris is healthy in appearance, but much more distant from the neural surface of the cornea than ordinary, and its mobility is less active, and more limited, than it ought to be. There is no inflammation of the eye present.

*Operation.* In this case I was desirous of operating upon one eye first, and of witnessing its effects, in order that I might be able to determine the probable advantage he might derive from the removal of the opaque capsule; for, as I shall presently more particularly explain, the retina is often, in these cases, permanently unfitted for the purposes of vision, and is not improved, is not altered in the slightest degree, when the original obstacle to perfect vision is taken away.

As an experiment, which could do no material injury, I introduced a small cataract-needle behind the margin of the cornea, as for the posterior operation of solution, removed the capsule from the pupil by depressing the point of the needle, so as to leave that part perfectly clear, and endeavoured to detach the opaque membrane from the iris, to which it had formed some adhesions. However, on withdrawing the instrument from his eye, the capsule quickly returned to its original situation. As this operation proved quite unsuccessful, I practised a different one about nine days afterwards: it consisted in making a small puncture in the cornea, introducing a pair of fine forceps within the incision, and beneath the corneal flap, and removing, or rather endeavouring to remove, the tough capsule; but I found that the adhesion of this membrane to the iris was of so firm a nature, that, rather than separate or destroy it, I could detach the iris from its ciliary connexion; I was content therefore to pull it through the pupil, and leave it in the anterior chamber. Soon after the operation had been performed, the opaque capsule retired into the posterior chamber. As soon as the very slight inflammation produced by this operation had subsided, I made an incision in the cornea with Beer's knife, and divided it pretty much as for the ordinary operation of extraction, (except, of course, that the division of the cornea was rather less extensive;) I then introduced a small hook behind the margin of the capsule, and withdrew it without the slightest difficulty. The eye was, in the course of a few days, in the state in which it is at the present time.

Last Wednesday I performed a similar operation upon the right eye only, dividing the cornea at its upper instead of at its lower part, and had no difficulty in removing the opaque capsule; and you have now an opportunity of seeing the benefits this operation has conferred. You may perceive that the pupil is quite clear and circular, that the iris is uninjured, and that no part of the opaque capsule remains.

Such, gentlemen, are the particulars of this case; and I will now proceed to offer a few general remarks on congenital cataract, and will conclude my observations by pointing out various circumstances connected with this case which have appeared to me to render it one of great practical interest.

You are aware that it is not now the custom to allow congenital cataract to remain, as happened in this case, without any attempts being made to effect its cure: it is established and correct practice to operate upon infants at the early age of from six to eighteen months;* and to Mr. Saunders is due, I believe, the credit of first distinctly recommending and bringing this practice into general notice; but I may mention that the friends of the late Mr. Gibson, of Manchester, claim for him the honour connected with this improvement in an important department of ophthalmic surgery.† Unquestionably it is a great improvement in the management of these cases not to wait, as was formerly the custom, until the subject of the disease had arrived at adult age, but to operate whilst the patient is very young; and I may tell you that, without mentioning the loss of time the continuance of the disease until adult age involves, that many serious evils occur in the structure and functions of the eye, and its ap-

* Mr. Lawrence recommends this operation to be performed earlier; at a period after birth varying from one to two months. (See his Surgical Lectures.) Dr. Farre says, that the greatest success attended Mr. Saunders' operations for congenital cataract, when performed on children between eighteen months and four years old.

† In the year 1801, Mr. Ware published an account of a case in which he succeeded in curing congenital cataract by the operation of solution, in a boy seven years old, and he then pointed out the propriety of extracting the capsule by an incision in the cornea, if it should be very thick and tough; in 1805, he repeats this suggestion, and again recommends the posterior operation of solution as the best means of curing congenital cataract; and in 1811 he states, that he has been in the habit of operating upon infants who had congenital cataract, by slightly lacerating the anterior capsule. (369.) Mr. Cheselden operated upon "young children," when born with cataracts, as appears from his statement contained in his small work on Anatomy, published in 1792, page 304.

pendages, which are distinctly referrible to the absence, on the part of the retina, of its due impressibility by light; and I am glad that the case before us gives me an opportunity of shewing you the more important of them.

In the first place, you will notice that the eye is remarkably small, that it is imperfectly developed. You are aware that if an organ be insufficiently exercised in a proper manner, and at a proper period, it is not duly and fully developed. This is in accordance with an established law of the animal economy, and you will find the investigation of this subject replete with interest; but it is sufficient for my present purpose to draw your attention to the fact in this very cursory manner, in order that I may point out to you how admirably it is illustrated as respects the development of the eye, when its functions are never properly called into exercise, in the case before us. You may perceive (and it is a point well worthy your attention), that although no part of the eye is wanting except the lens and its capsule—that, with these exceptions, it is perfect as respects the *number* of its parts; and that, although its various parts are all of them actually existent and evolved, yet the organ is exceedingly small; the actual size of the eye is in fact so much less than usual, that its diminutiveness is palpable to common observation.*

The next circumstance to which I shall direct your observation, is the unsteady, rolling motion of the eyeball. You remark that, when a person is desirous of looking attentively at an object, his eye becomes steady and fixed; such a state of the eye is essential to the accurate perception of minute objects, to what may be termed minute vision. But, if you examine the eyes of an adult in whom congenital cataract exists, you observe that they are continually darted in various directions; the muscles of the globe have lost their steadiness of action, and have become in a great measure independent of the will. This rolling, unsteady, and vacillating motion of the eyeball is clearly seen in the present case. It appears that the retina does not receive a distinct impress from any one object; no impress sufficiently decided to engage the powers of vision in the definite and determined contemplation of its characters.

Another circumstance will, I am sure, have occurred to your minds, as relates to the absence of the natural stimulus

* I have lately had an opportunity of seeing two young persons who were born with cataract in each eye, and in whom the hands and feet were exceedingly small. This imperfection of development was so obvious, that a professional friend, who saw these persons with me, remarked that they appeared like the hands and feet of an infant attached to the limbs of an adult. I have several times observed various errors in the formation and development of different parts of the system in persons suffering from congenital cataract.

of the retina during a long term of years; it will become torpid, and its susceptibility will be diminished. You may now observe this fact, and you will find, on making inquiry, from the subject of these clinical remarks, that, although the membranous cataract is removed, the pupil rendered clear, and, in short, although every mechanical impediment to the free transmission of light is taken away, he cannot see so well as those persons generally do who are cured by the usual operation of those forms of cataract which occur in advanced life. Your physiological knowledge will have caused you to anticipate such a result; but I need not enter upon the consideration of those circumstances which determine the degree of perfection with which the different organs perform their various functions.

Such, then, are some of the necessary consequences of the long continuance of congenital cataract, namely, 1, imperfect development of the eye; 2, a constant, unsteady, rolling or vacillating motion of the globe; and 3, impaired susceptibility of the retina: and you will remember that the two first of these injurious effects are never afterwards materially relieved, and that the latter is very generally a permanent defect; that is, although the sensibility of the retina may be much increased after the obstacle to the free transmission of light is taken away, yet its sensibility is even then much less than that of a perfectly-healthy eye. To the same effect is the testimony of Dr. Farre, in allusion to the practice of the late Mr. Saunders: he says, the sensibility of the retina, "in many of the cases cured at the ages of four years and under, could not be surpassed in children who had enjoyed vision from birth; but at eight years, or even earlier, the sense was evidently less active; at twelve, it was still more dull; and, from the age of fifteen and upwards, it was generally very imperfect, and sometimes the mere perception of light remained."

If congenital cataract be allowed to remain without any efforts being employed for its cure, the opaque capsule (the usual remnant of congenital cataract), is apt to acquire adhesions to surrounding parts, and particularly to the iris; and this circumstance very much diminishes the chances of restoring unimpaired vision, besides adding greatly to the difficulties associated with the performance of an operation for its cure.

Of course, there are other evils connected with the long continuance of congenital cataract, as its necessary effects, but they are of a minor character, and I am unwilling to occupy your time in pointing them out in detail.

You may imagine that congenital cataracts vary in their colour and consistence, and also as to the part which is the seat of the opacity. I shall explain to you what *generally* occurs, and shall then mention to you the real state of disease in the case of the young man whose eyes you have just examined. The lens is generally of a whitish colour, and often presents a milky appearance; it is very soft, almost fluid, and the capsule is transparent. This is, in nine cases out of ten, the exact nature of the *original* disease, as it exists during the first years of life; but, in the course of a few years, the lens becomes absorbed, the capsule becomes opaque, and its two layers, not being separated by the intervention of the lens, unite and form one tough membrane, such as you may observe in the preparation I now pass round. This preparation-glass contains the two tough, opaque, circular membranes, which I removed, as you know, from the eyes of Thomas Gardner, and which are, in fact, the united hemispheres of the capsule of the lens. You will understand that, in accomplishing this union of the two layers of the capsule, that it is the anterior capsule which retires upon the posterior as the lens becomes absorbed; for the latter, (the posterior capsule,) in consequence of its firm connexion with the hyaloid membrane, does not change its situation.

It is a question whether or not the opaque capsule possess the power of absorbing the lens, and I am disposed to believe that it does not possess any such capacity.* You must carefully notice those cases in which there exists an opacity of the capsule, the lens remaining, and observe whether or not it becomes absorbed after the capsule is thoroughly opaque; but I may remind you, in conducting this inquiry, to determine previously, as correctly as you can, the state of the posterior capsule, and to decide how far absorption may go on through the texture—not by the agency of the lenticular surface of the capsule. Attention to these two circumstances is absolutely essential, to enable you to arrive at any approach to a correct conclusion upon the subject. I have never seen an opacity of the capsule of the lens as a congenital disease, the lens itself remaining transparent; nor have I observed the existence of a hard congenital cataract in very early life.

Now, respecting the mode in which this form of congenital cataract becomes eventually membranous, I am desirous that you should thoroughly understand the nature of the

* The absorbing power of serous membranes generally is always impaired, and sometimes totally destroyed, when rendered densely opaque by disease.

process by means of which this conversion of disease is effected. Let me then repeat, that the lens is first opaque; that, as soon as the opaque lens becomes absorbed, the capsule which absorbed it becomes opaque; that the two layers of the capsule eventually coalesce, and very frequently acquire adhesions to the iris. Hence you perceive the reason why this thick, tough, opaque membrane is not readily removed by the same process which causes the absorption of the lens. Mr. Saunders alludes to this point in very decided terms, and he says that, if this tough capsule be allowed to remain, it can neither be *depressed* nor *extracted* (156;) and he recommends that an attempt should be made to form an opening with a needle in the centre of the tough capsule. Now you will find, on a careful examination of this subject, that Mr. Saunders had some grounds for the expression of this opinion; but they were delusive, and the reasoning founded on them was quite erroneous. If the thick capsule be allowed to remain for a long period before an attempt be made to remove it, it acquires (I am speaking of the general rule), adhesions to the iris, and is very often strongly connected with the hyaloid membrane; and the former of these circumstances much increases the difficulty of extracting, and the latter of depressing, it; and you know that, in relating the case which forms the basis of my remarks this evening, I told you that when I had removed the capsule from the pupil by my first operation, it afterwards recovered its original situation; and this, you will perceive, took place in consequence of the firm adhesion subsisting between the capsule and the hyaloid membrane; and you will further remember that, in consequence of the small size of the incision of the cornea, I could not, at my second operation, move with sufficient freedom the instruments necessary to destroy the adhesion subsisting between the iris and the capsule, and that, if I had persisted in my attempts to withdraw that membrane through the opening in the cornea, I must necessarily have torn the iris from its ciliary attachment. If I succeeded in removing the tough capsule most readily at my third operation* on the left eye, and also at my first operation upon the right eye, it was chiefly because I had made the incision of the cornea sufficiently large to enable me to move the hook and forceps very freely, when introduced beneath the corneal flap. And here, in justice to the memory of the late Mr. Gibson, of Manchester, who was a most talented and enthusiastic culti-

* The previous operations had destroyed some, and loosened other, adhesions of the capsule to the iris, so as to assist in enabling me to remove it the more easily at my third operation.

vator of our interesting profession, I should tell you that we are indebted to him for proposing,—or, I should rather say, for urgently and distinctly recommending, the removal of tough capsular cataract by a section of the cornea.

Mr. Gibson pointed out that very little inflammation was excited by the simple section of the cornea, and by certain manipulations necessary to effect the removal of the opaque capsule in cases similar to that to which I am now directing your attention; but you will be aware that, unless you can remove the capsule pretty quickly, and without the frequent introduction of instruments, your manœuvres will be likely to give rise to great inflammation of the iris, or of the textures of the eye generally; and you will also be aware of the difficulty of using instruments in so small a space as that which is left by the edges of the incision you have made in the cornea; and I presume that a practical acquaintance with these difficulties suggested to Mr. Gibson the necessity of contriving those beautiful instruments, Gibson's hook, forceps, and scissors, the invention of which was original as respects Mr. Gibson.

You will remember that Mr. Gibson made merely a small puncture of the cornea, but I have been foiled on several occasions in consequence of adhering to his advice in this respect; and knowing that the cornea may be pretty freely divided in persons who are neither very young nor very old, without hazarding the occurrence either of sloughing, or any material degree of inflammation, I have thought it better to make (and in fact I have done so in my more recent operations), an incision of the cornea pretty much the same as for an ordinary operation of extraction; perhaps about one-fourth less as regards the whole extent of the section. Through an opening of this kind and extent, you can operate upon the opaque capsule with ease and freedom; and I am inclined to regard this alteration as a decided improvement upon the method proposed by Mr. Gibson. It must be admitted, as an objection to the more extended incision, that a puncture of the cornea is more readily made than the section of that membrane, as I have advised; but this objection will only exist in reference to your earlier operations, for a little practice will render either of these operations easy of performance. And here I may remark, that, to assist you in fixing the eye before making the puncture of the cornea, the index and middle fingers of the left hand, (if you are operating upon the left eye,) must project beyond the tarsal margin of the lower lid, the middle finger should be pressed firmly against the eyeball towards its nasal side, and the indexfinger must be pressed in the same manner against the globe at its temporal side, so as to steady the eye; and you may employ more force in doing so than in an ordinary operation for extraction; for I can tell you, that the rolling, jerking, unsteady movements the eye has acquired, will render it a matter of great difficulty to divide the cornea, unless you adopt this or some other mode of controlling them.

If you have an opportunity of seeing a case of congenital cataract in an infant, you would, of course, recommend an operation for its cure without delay, if it be in good health, and more than six months old; and, under such circumstances, you would not think of dividing the cornea, and endeavouring to extract the cataract, but you would prefer the posterior operation of solution, as advised by Ware, Gibson, and Saunders; and, on the contrary, if your advice is requested respecting a case of congenital cataract, such as existed in the young man you have seen this evening, you would extract it in the way I have recommended, which is, you know, a modification of the method first suggested by Mr. Ware, but more particularly and precisely recommended, described, adapted, and practised by the late Mr. Gibson; and, if any firm and extensive adhesions exist between the tough capsule and the iris, you would introduce Gibson or Maunoir's scissors beneath the corneal flap, and make an aperture in the opaque capsule, and cut it away as nearly as possible on a level with the pupillary margin of the iris; the loose fragments of the capsule may then be withdrawn, either by means of the small hook or the forceps.

If the cataract, which was originally lenticular, become membranous, and the two layers of the capsule occupy the situation of the pupil, and become united, you ought not to attempt its cure by the operation of solution, because the toughness and compactness of its texture renders it exceedingly difficult of solution, and very little under the influence of the absorbents; neither ought you to attempt to depress it; for the firmness of its adhesion to the hyaloïd membrane renders that operation very difficult of performance: indeed, it is frequently attempted in vain. For this state of things the operation of extraction is decidedly preferable; but if the cataract occur in infancy, the operation of extraction would be unnecessary and unadvisable; unnecessary, because the case may be readily cured by means of a less difficult and important operation; and unadvisable, because, at the period of infancy, the iris is very near to the cornea, and the cornea is thicker, its texture is looser, and its lamellæ are more moveable upon each other, than in advanced life; so that it is extremely difficult to divide the cornea under such circumstances, without either wounding and injuring the iris, or leaving behind extensive opacity of the cornea. You are, I apprehend, familiar with the anatomical characters of the cornea in infancy, as compared with its qualities in the middle and subsequent periods of life, so minutely described by Zinn, Clemens, and Soëmmering.

Now I have not entered into a minute description of the mode in which I performed the operation in the case of Thomas Gardner, for, as many of you witnessed the operation, I have not thought it necessary to do so; and, besides, I have been chiefly anxious to direct your attention to the circumstances which would determine the propriety of selecting some particular operation for the cure of congenital cataract, according as it may exist in early or in more advanced life. I have also purposely omitted to speak of the adaptation of glasses to that condition of the eye which remains after the operation, and of the patient's notions of the distances, colours, dimensions, and forms of objects, on acquiring the increased vision which an operation, performed for the cure of the congenital cataract at a certain age, is so well calculated to confer.*

In concluding these remarks, I wish to state that I shall not fail to continue the occasional delivery of clinical lectures, whenever those circumstances exist which, as I have already explained, have induced me to appear before you on the present occasion; at least, so long as you evince a desire to attend them.

* See the observations relating to this interesting subject, by Cheselden, Ware, Wardrop, Maunoir, Dupuytren, &c.

